# Polymorphism of *leptin* gene (single nucleotide polymorphisms c.73T>C) and its association with body weight and body measurements in Madura cattle

**DOI:** 10.14202/vetworld.2022.775-781

**Published:** 2022-03-30

**Authors:** Kuswati Kuswati, Ahmad Furqon, Wike Andre Septian, Trinil Susilawati

**Affiliations:** Department of Animal Production, Faculty of Animal Science, Universitas Brawijaya Jl. Veteran, Malang 65145, Indonesia

**Keywords:** body measurements, leptin, Madura cattle, polymorphism

## Abstract

**Background and Aim:**

Madura cattle is local cattle in Indonesia. This cattle hasphenotypic variations in growth traits. This study aimed to identify *leptin* (LEP) gene polymorphism exon 2 associated with body measurements in Madura cattle.

**Materials and Methods:**

We recorded body weight (BW) and body measurements of 51 Madura cattle aged 1-4 years in Waru District, Madura. The LEP gene genotyping was conducted using the polymerase chain reaction-restricted fragment length polymorphism method with *Aci*I restriction enzyme.

**Results:**

A 267 bp DNA fragment of the LEP gene was successfully amplified using a pair of primers. This study revealed three genotypes (TT, TC, and CC) and two alleles (T and C). The frequencies of TT, TC, and CC genotypes were 0.275, 0.45, and 0.275, respectively, whereas the frequencies of T and C alleles were 0.500 and 0.500, respectively. The c.73T>C mutation was significantly associated with BW, body length (BL), and chest girth (CG) (p*<*0.05). Among all genotypes, the TC had the highest BW, BL, and CG.

**Conclusion:**

Conclusively, LEP gene polymorphism (c.73T>C) exon 2 was polymorphic and associated with body measurements, especially BW, BL, and CG.

## Introduction

Livestock, an agricultural subsector, plays a crucial role in providing food as a protein source for people. The livestock product demand is increasing because of the rapid human population growth. This demand can be met by increasing livestock population and meat production, particularly beef [1]. Madura cattle are local cattle breeds that originated from Madura Island, Indonesia. Madura cattle are a crossbreed of Bali cattle (*Bos javanicus*) and Zebu cattle (*Bos indicus*) [2]. Farmers commonly raise Madura cattle for various purposes, such as beef cattle, bull racing (Karapan), and cow contest (Sonok) [3]. Madura cattle have several advantages, including high carcass percentage, good meat quality, and good adaptability in rural environments [1,2,4]. Furthermore, Madura cattle can adapt to low feed quality and are resistant to some diseases and tick infestation [5]. In general, Madura cattle exhibit phenotypic variation, especially in growth traits [6].

Several factors, including genetics, nutrition, and the environment, affect growth traits [6,7]. Many genes have been identified to regulate the growth traits in cattle reported in the previous studies, such as growth hormone (GH), growth hormone receptor (GHR), insulin-like growth factor 1 (IGF1), IGF1 receptor, *leptin* (LEP), and leptin receptor [8-11]. LEP is a hormone predominantly produced by white adipose tissue and secreted into the bloodstream [12,13]. This hormone plays a critical role in controlling body weight (BW), feed intake, energy metabolism, fat deposition, immune function, and reproduction [14,15]. LEP hormone is encoded by the LEP gene [16]. The previous studies [16,17] described association analyses between single nucleotide polymorphisms (SNPs) of the LEP gene. The SNP c.73T>C in LEP gene exon 2 is associated with growth rate and fatness [17]. The SNP has the potential to develop as a genetic marker for growth traits [17].

Regarding the statement mentioned above, genetic improvement can be achieved using appropriate techniques, such as a well-designed selection program [16,18,19]. The genetic marker in the LEP gene (SNP c.73T>C) can be used to support the success of the selection program. Unfortunately, data on SNP c.73T>C in Madura cattle are limited. Therefore, this study was designed to identify the LEP gene polymorphism exon 2 associated with BW and body measurements in Madura cattle.

## Materials and Methods

### Ethical approval

The Animal Care and Use Committee of Universitas Brawijaya (No. 019-KEP-UB-2021) approved all procedures in this study.

### Study period and location

This study was conducted from April 2020 to October 2020 at Village Breeding Center (VBC) in Waru, Pamekasan, Madura and Laboratory of Animal Biotechnology, Faculty of Animal Science, Universitas Brawijaya, Malang, Indonesia.

### Data collection and animal management

In this study, 51 female Madura cattle aged 1.5-4 years were used. The age of cows was determined by the eruption of permanent incisor (PI) teeth starting from PI0 to PI8. Blood sample (3 mL) from each animal was collected from the *Vena jugularis* using vacutainer needles and ethylenediaminetetraacetic acid tubes. Blood samples were immediately stored in a icebox (4°C) and transferred to a freezer at −20°C [16]. The BW and body measurements of the cows were obtained from a VBC in Waru District, Madura [7]. The cattle were from one sire. This sire was specifically needed in the VBC as superior male cattle known as Adikara. Madura cattle used in this study were limited from selected cows for the cultural contest, namely Sonok. Body measurements included withers height (WH), body length (BL), chest girth (CG), hip height (HH), head length (HL), and head width (HW) from cattle [7]. The cows were raised traditionally, with a similar feeding system and management. The cows had free access to water and were housed in groups.

### DNA isolation and polymorphism genotyping by polymerase chain reaction-restriction fragment length polymorphism (PCR-RFLP)

Genomic DNA was isolated using Genomic DNA Mini Kit for blood and cultured cell (Geneaid Biotech Ltd., China). The DNA isolation procedure was conducted following the manufacturer’s instructions. The quality of DNA was checked using 1.5% agarose gel electrophoresis, whereas the quantity was measured using NanoDrop ND-1000 Spectrophotometer (Thermo Fisher Scientific™, Massachusetts, USA) [17].

A 267 bp DNA fragment of LEP gene exon 2 was amplified using the PCR method with a pair of primers. The forward primer is 5′-CAT CTG AAG ACG TGG ATG CG-′3, and the reverse primer is 5′-CCT ACC GTG TGT GAG ATG TC-′3. The primers were designed on the basis of the bovine genomic sequence in GenBank (accession number U50365.1). PCR was conducted in a 30 mL volume containing DNA template (50-100 ng/mL), primers (10 pmol/mL), 1× Go Taq Green Master Mix (Promega, USA), and Nucleus Free Water (NFW). The PCR machine conditions of Bio-Rad T100™ Thermal Cycler (Bio-Rad, USA) were set for a predenaturation temperature at 94°C for 5 min, 35 cycles of 94°C for 10 s (denaturation), 60°C for 20 s (annealing), 72°C for 30 s (extension), and a final extension at 72°C for 5 min. Furthermore, the PCR products were checked using electrophoresis for 35 min at 100 V on 1.5% agarose gel. The agarose gel was stained using Diamond™ Nucleic Acid Dye (Promega) and visualized under the blue light of Glite 965 GW imaging system (Pacific Image Electronics Co., Ltd., Taiwan) [17].

The PCR product was digested by a restriction enzyme in a 7 μL volume containing 5 μL of PCR product, 0.7 μL of 10× reaction buffer, four units of *Aci*I (ER1791, Thermo Fisher Scientific), and 0.9 μL of NFW. The digested products were separated by electrophoresis in 2% agarose gel, visualized under the blue light of Glite 965 GW imaging system, and analyzed to determine the genotypes on the basis of the DNA band patterns. The genotypes were confirmed by targeted DNA sequencing at 1^st^ BASE DNA Sequencing Services in Selangor, Malaysia [17].

### Statistical analysis

Gene polymorphism was analyzed by calculating the frequency of genotype and allele and Hardy–Weinberg equilibrium. This study adopted a randomized complete block design. The fixed factor was the genotypes, whereas the age (i.e., blocks) was randomly selected from the cattle population. The dependent variables include BW, withers height (WH), BL, CG, HH, HL, and HW. The association study between LEP gene polymorphism and BW and body measurements was analyzed using the General Linear Model procedure in SPSS ver. 26.0. The mathematical model used in this research was

Y_ijk_=μ+G_i_+A_j_+ɛ_ijk_

where Y_ijk_ is the BW and body measurements of j^th^ cows, i^th^ genotype, and k^th^ age; μ is overall mean; G_i_ is the effect of i^th^ genotype; A_k_ is the effect of k^th^ age; and ɛ_ijk_ is the random error [7].

The null hypothesis: There was no association between LEP gene polymorphism and BW and body measurements in Madura cattle.

The alternative hypothesis: There was an association between LEP gene polymorphism and BW and body measurements in Madura cattle.

## Results

### Madura cattle phenotype

In this study, BW and body measurements of Madura cattle were obtained at PI0, PI2, PI4, PI6, and PI8 of age. Age did not significantly affect the BW, WH, BL, CG, HH, HL, and HW (Table-1). Table-2 shows the general description of BW and body measurements. Madura cows had a mean BW of 283.53, a mean WH of 121.76 cm, a mean BL of 126.65 cm, a mean CG of 156.24 cm, a mean HH of 122.18 cm, a mean HL of 39.76 cm, and a mean HW of 17.10 cm.

**Table-1:** Body weight and body measurements grouped by age.

Variables	PI0 (n=18)	PI2 (n=6)	PI4 (n=16)	PI6 (n=3)	PI8 (n=9)	Significance
BW (kg)	272.61±74.72	305.83±42.06	298.00±62.27	274.67±24.58	269.38±49.02	NS
WH (cm)	120.06±8.73	127.50±4.28	121.93±6.25	124.33±8.74	120.22±5.09	NS
BL (cm)	123.06±11.49	132.67±3.93	127.33±7.23	128.33±7.51	128.11±7.99	NS
CG (cm)	153.72±15.43	160.00±9.06	160.87±10.84	154.67±2.08	151.56±10.99	NS
HH (cm)	120.11±8.87	125.67±5.65	122.47±6.45	121.67±3.79	123.67±6.34	NS
HL (cm)	39.50±3.59	41.83±2.64	39.47±2.33	39.67±3.21	39.44±5.96	NS
HW (cm)	16.56±1.62	16.83±1.72	18.00±2.51	17.00±2.65	16.89±2.15	NS

PI0=Permanent incisor 0, PI2=Permanent incisor 2, PI4=Permanent incisor 4, PI6=Permanent incisor 6, PI8=Permanent incisor 8; n=Number of samples, BW=Body weight, WH=Withers height, BL=Body length, CG=Chest girth, HH=Hip height, HL=Head length, HW=Head width, NS=Not significant (p>0.05)

**Table-2:** Sample size, mean, SD, and range of traits in Madura cattle.

Variables	n	Mean	SD	Minimum	Maximum
BW (kg)	51	283.53	61.59	113.00	464.00
WH (cm)	51	121.76	7.19	95.00	134.00
BL (cm)	51	126.65	9.12	100.00	144.00
CG (cm)	51	156.24	12.49	115.00	189.00
HH (cm)	51	122.18	7.21	90.00	136.00
HL (cm)	51	39.76	3.64	25.00	45.00
HW (cm)	51	17.10	2.08	12.00	21.00

n=Number of samples, BW=Body weight, WH=Withers height, BL=Body length, CG=Chest girth, HH=Hip height, HL=Head length, HW=Head width, SD=Standard deviation

### LEP gene polymorphism

**Figure-1:**
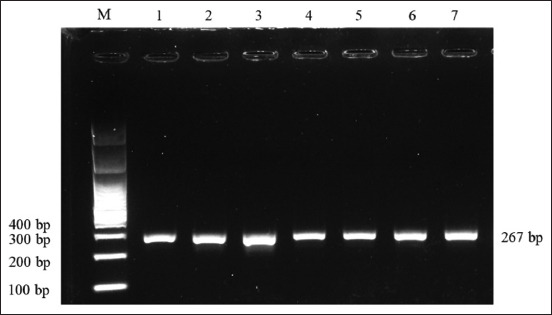
Visualization of polymerase chain reaction product on 1.5% electrophoresed agarose gel, M: 100 bp marker, 1-7: samples.

**Figure-2:**
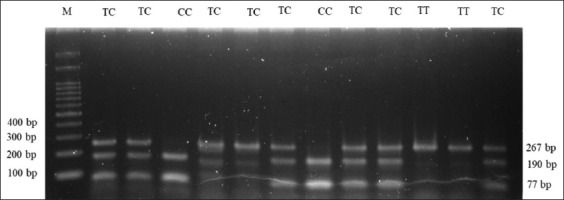
Visualization of digested polymerase chain reaction product by *Aci*I enzyme on 2% electrophoresed agarose gel, M: 100 bp marker.

**Figure-3:**
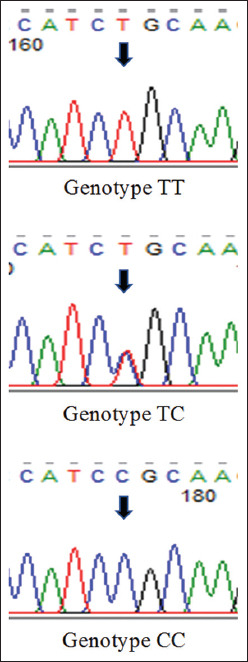
The chromatograph of *Leptin* genotypes (c.73T>C).

In this study, the SNP c.73T>C of the LEP gene was investigated. DNA fragments of the LEP gene on 51 Madura cows were successfully amplified using PCR at 60°C annealing temperature. The 267 bp of PCR product was detected and visualized on 1.5% electrophoresed agarose gel (Figure-1). The PCR product was digested by the *Aci*I restriction enzyme. Three genotypes of the LEP gene were determined by the patterns of digested PCR products on 2% electrophoresed agarose gel (Figure-2) and confirmed by targeted DNA sequencing (Figure-3). The TT genotype had a DNA fragment length similar to the PCR product, which was 267 bp. The CC genotype had two fragments (190 and 77 bp), whereas the TC genotype had three fragments (267, 190, and 77 bp).

In this study, the frequencies of TT, TC, and CC genotypes were 0.275, 0.450, and 0.275, respectively (Table-3). Two alleles were detected, namely, T and C. The frequency of the T allele was 0.500, which was equal to the frequency of the C allele. According to the allele frequency, the SNP c.73T>C of the LEP gene exon on Madura cattle was polymorphic. The population of Madura cattle in the VBC Waru was in Hardy–Weinberg equilibrium because of a lower *Χ*^2^ value than *Χ*^2^ (0.05;1) at 3.84.

**Table-3:** Frequencies of genotypes and alleles of the LEP gene (c. 73T>C) in Madura cattle.

Breed	n	Genotype frequencies			Allele frequencies		χ^2^ value
	
		TT	TC	CC	T	C	
Madura cattle	51	0.275	0.450	0.275	0.500	0.500	0.49^ns^

n=Number of samples; ns=Not significant, χ^2^ (0.05;1)=3.84, LEP=*Leptin*

### Effect of LEP genotypes on BW and body measurements

The association study was conducted for SNP c.73T>C with BW and body measurements in Madura cattle (Table-4). In this study, SNP c.73T>C was significantly associated with BW, BL, and CG (p*<*0.05). The TT genotype of SNP c, 73T>C had a lower mean BW and CG than the TC genotype (p*<*0.05). The BL of the TT genotype was the lowest among all genotypes (p*<*0.05). Even though the SNP c.73T>C did not significantly affect (p>0.05) WH, HH, HL, and HW, the TT genotype showed the lowest WH, HH, HL, and HW, descriptively.

**Table-4:** Association of LEP genotypes with body weight and body measurements.

Variables	TT (n=14)	TC (n=23)	CC (n=14)	p-value
BW (kg)	256.86±15.65^a^	307.5±12.48^b^	271.69±16.24^ab^	0.036
WH (cm)	118.50±1.85	124.13±1.44	121.14±1.85	0.062
BL (cm)	120.07±2.22^a^	129.83±1.73^b^	128.00±2.22^b^	0.004
CG (cm)	150.86±3.13^a^	161.57±2.44^b^	152.86±3.13^a^	0.017
HH (cm)	118.86±1.88	123.74±1.47	122.93±1.88	0.122
HL (cm)	39.00±0.97	40.57±0.76	39.21±0.97	0.366
HW (cm)	16.14±0.54	17.78±0.42	16.93±0.54	0.060

n=Number of samples, BW=body weight, WH=withers height, BL=body length, CG=Chest girth, HH=hip height, HL=head length, HW=head width. Means within the same row with different superscripts are significantly different (p<0.05), LEP=*Leptin*

## Discussion

Regarding the phenotypic aspect, the varying ages of Madura cattle did not significantly affect BW and body measurements in this study. In ruminant animals, BW and body size increase with age until mature age in young animals [7,20]. The difference between age groups in this study may be due to using a small number of animals or the fact that the research was conducted on adult animals that completed their growth. This study revealed that Madura cattle had a BW of 283.53 kg, a WL of 121.76 cm, a BL of 126.65 cm, and a CG of 156.24 cm. A previous study reported that adult female Madura cattle had a BW of 282.76 kg, a WH of 123.42 cm, a BL of 127.02 cm, and a CG of 155.79 cm. Another study reported that young Madura cattle had a BW of 182.86 kg, a WH of 108.69 cm, a BL of 110.81 cm, and a CG of 133.41 cm [21]. Regarding the larger BW and body measurements in this study, it might be caused by the location in the VBC of Madura cattle. In this area, most Madura cattle were raised for the cattle contest.

LEP is a polypeptide hormone produced by adipose tissue and expressed by skeletal muscle, mammary gland, and other tissues [16,22]. This hormone plays a crucial role in controlling feed intake by inhibiting hunger and acting on the specific receptor (LEP receptor) in the hypothalamus, dorsomedial, and arcuate nuclei [22]. LEP also plays a role in providing information about body fat deposition to the brain [23]. The increase in adipose tissues triggers the release of more LEP to the bloodstream because of the increase in BW, and this will be detected by brain receptors [24]. Puberty affects the maturity of the reproductive system, and it will occur when the body’s energy store is in adequate condition [25].

LEP hormone is encoded by the LEP gene. The bovine LEP gene is located in BTA 4q32 and consists of three exons and two introns [26]. The previous studies have associated SNP (c.73T>C) of LEP gene exon 2 with BW, fatness, and carcass quality [27,28]. This SNP is defined as a nonsynonymous mutation that changes amino acids from cysteine to arginine. Other SNPs of the LEP gene (g.1025T>C and g.1048G>A) were also reported in a previous study [29].

In this study, the SNP c.73T>C polymorphism was successfully identified using PCR-RFLP with *Aci*I restriction enzyme (5-C|CGC-3). PCR-RFLP was widely used to identify the gene polymorphism associated with economic traits of animals, such as body compositions, lactation, reproduction, immune system, fatty acids composition, carcass, and meat quality [30-33]. This study revealed three genotypes of TT, TC, and CC. The frequencies of TT, TC, and CC allele on Madura cattle were 0.275, 0.450, and 0.275, respectively. The present findings indicated that most Madura cattle had TC genotype and the frequencies of T and C allele were equal (0.500 for each allele). By contrast, most previous studies have reported that the frequency of the C allele was higher than that of the T allele in Kebumen Ongole Grade, Brahman, and Turkey cattle [10,16,26,]. In addition, the frequencies of the C allele and CC genotype were highest in Nellore cattle [34]. The differences in LEP gene polymorphism might be due to differences in gene expression among the cattle breeds [26]. Based on this variation of the LEP gene, a molecular selection program could be performed to determine the Madura cattle with good growth and body measurements.

Regarding the nonsynonymous mutation of c.73T>C, the population of Madura cattle in VBC Waru was in Hardy–Weinberg equilibrium. A similar result was reported by Fernandes *et al*. [35] in the Nellore cattle population. This equilibrium occurred when the Chi-square value was smaller than the Chi-square table [36]. When the population is in Hardy–Weinberg equilibrium, the allele and genotype frequencies will remain the same across generations, indicating that random mating probably occurred and there is no genetic change caused by any factors in the population. The change in allele frequencies was caused by some factors, such as mutation, inbreeding, gene recombination, genetic drift, and gene flow from other populations [37].

This study discovered the association between LEP gene polymorphism and BW and body measurement. The SNP c.73T>C polymorphism significantly affected BW, BL, and CG (p*<*0.05). The TC genotype had a higher BW and BL than the TT genotype. The TT genotype had the lowest BW, whereas the TC genotype had the highest CG. In Nellore cattle, the animal with TC genotype had higher BW and average daily gain [38]. A previous study reported that TC genotype was superior in weaning chest circumference on Kebumen Ongole Grade cattle [26]. Another study reported that the TC genotype had higher reproductive efficiency and age at first calving in Nellore heifers [35]. By contrast, the TC genotype had the lowest ovary length for reproductive traits on commercial beef heifers [39]. Regarding the substitution of amino acids from cysteine to arginine, it may alter the biological function of LEP, especially the protein molecular function. The presence of cysteine in an LEP molecule might interfere with LEP binding to its receptor, which could disrupt the stability of disulfide bonding [29]. LEP binds to its receptor on neuropeptide Y-neurons, resulting in increased energy output and hypophagia [40]. Furthermore, the extra cysteine to proteins results in the loss of protein biological function because of the presence of the T allele of SNP c.73T>C [41]. It could probably explain that the TT genotype had the lowest BW and body measurements in Madura cattle.

## Conclusion

An association study of LEP gene exon 2 polymorphism and BW and body measurements was successfully conducted in Madura cattle. A novel finding revealed that the TC genotype has a high BW, BL, and CG in Madura cattle. This study supports the candidacy of the LEP gene for further research as a potential genetic marker in the selection program of Madura cattle.

## Authors’ Contributions

KK: Designed the study, performed experimental work, conducted a literature review, interpreted the data, drafted and proofread the manuscript, and served as the project advisor. AF: Designed the study, performed data analysis, interpreted the data, and reviewed the manuscript. WAS: Performed experimental work and conducted a literature review. TS: Designed the study, served as the project advisor, and reviewed the manuscript. All authors read and approved the final manuscript.
